# Regulating Inflammation Using Acid-Responsive Electrospun Fibrous Scaffolds for Skin Scarless Healing

**DOI:** 10.1155/2014/858045

**Published:** 2014-03-25

**Authors:** Ziming Yuan, Jingwen Zhao, Yigang Chen, Zhili Yang, Wenguo Cui, Qi Zheng

**Affiliations:** ^1^Department of General Surgery, Shanghai Sixth People's Hospital, Shanghai Jiao Tong University, School of Medicine, 600 Yishan Road, Shanghai, 200233, China; ^2^Orthopedic Institute, Soochow University, 708 Renmin Road, Suzhou, Jiangsu, 215006, China; ^3^School of Biomedical Engineering and Med-X Research Institute, Shanghai Jiao Tong University, Shanghai, 200030, China; ^4^Department of Orthopedics, The First Affiliated Hospital of Soochow University, 188 Shizi Street, Suzhou, Jiangsu, 215006, China

## Abstract

Skin injury in adult mammals brings about a series of events and inflammation in the wounded area is initiated first and provides lots of inflammatory factors, which is critical for the final scar formation. While the postinjured skin of fetus and nude mice heals scarlessly owing to the absence of inflammation or immunodeficient, we designed a feasible acid-responsive ibuprofen-loaded poly(L-lactide) (PLLA) fibrous scaffolds via doping sodium bicarbonate to prevent excessive inflammation and achieve scarless healing finally. The morphological results of in vivo experiments revealed that animals treated with acid-responsive ibuprofen-loaded PLLA fibrous scaffolds exhibited alleviative inflammation, accelerated healing process, and regulated collagen deposition via interference in the collagen distribution, the **α**-smooth muscle actin (**α**-SMA), and the basic fibroblast growth factor (bFGF) expression. The lower ratios of collagen I/collagen III and TGF-**β**1/TGF-**β**3 and higher ratio of matrix metalloproteinase-1 (MMP-1)/tissue inhibitor of metalloproteinase-1 (TIMP-1) in acid-responsive ibuprofen-loaded PLLA fibrous scaffolds group were confirmed by real-time qPCR as well. These results suggest that inhibiting the excessive inflammation will result in regular collagen distribution and appropriate ratio between the factors, which promote or suppress the scar formation, then decrease the scar area, and finally achieve the scarless healing.

## 1. Introduction

Skin wound healing in adult mammals is a dynamic process, involving inflammation, proliferation, and maturation or remodeling phases [1]. Inflammation is initiated first, with neutrophil accumulation in the wound area, followed by resident macrophages and circulating monocytes infiltrating the wound [2]. Proliferation is characterized by reepithelialization, angiogenesis, granulation tissues formation, and matrix deposition [3] that inevitably leads to fibrotic scar tissue formation. Remodeling is the accumulation and remodeling of collagen, which is largely regulated by fibroblasts and forms scars finally. These scars may cause problematic results, particularly when wounds across joints, in which they can impair the mobility and flexibility or affect conspicuous locations, which can result in devastating psychological consequences [4]. In contrast to adult wound healing, fetal skin wounds healing without scars, to some extent, attributing to them do not mount an inflammatory response after injury until late in gestation [5]. In another study, the postinjured skin of nude mice is characterized by lack of scar, since nude mice are immunodeficient [6]. So control inflammation in the process is quite important for the final scarless healing.

In view of the scarless healing of the early fetus, there are several characteristics of regeneration without scars. For example, sufficient content of type III collagen may prevent scar tissue formation, and excessive secretion of type I collagen may result in a disorganized fiber structure and hypertrophic scar formation [7]. There are three highly homologous transforming growth factor-*β* (TGF-*β*) genes in mammals, TGF-*β*1, TGF-*β*2, and TGF-*β*3 [8]. In general, TGF-*β*1 and TGF-*β*2 are known to promote fibroplasia and scar, while TGF-*β*3 is believed to reduce scar [9–11] and downregulate TGF-*β*1 and TGF-*β*2 [8]. Matrix metalloproteinases (MMPs) constitute a family of zinc endopeptidases that are capable of degrading most of the structural components of the extracellular matrix (ECM) [12] and are regulated by their tissue inhibitors (TIMPs) [13]. Scarless wounds have a higher ratio of MMP to TIMP, favoring remodeling and less accumulation of collagen [14]. In particular, TGF-*β* can be secreted by neutrophils, lymphocytes, macrophages, and so on [15], which are critical in inflammatory response, and TGF-*β* can promote ECM synthesis and inhibit matrix turnover through the regulation of MMPs and TIMPs as well [16]. Thus, the regeneration without scar through regulating the collagen, TGF-*β*, MMP, and TIMP by controlling the inflammation desperately needs to be studied.

For the treatment of skin lesions, several strategies are currently available, such as, topical application of complement C5 [17] or microbial cellulose [18]. These methods speed the healing process of wounded skin, but they neglected the scar formation during this process. It has been reported that basic fibroblast growth factor (bFGF) has a possible antiscarring effect [19]. However, the bioactivity and bioavailability of bFGF applied in the wounded area are unwarrantable. Electrospinning is a widely used technique for the production of nanofibers from various natural or synthetic polymers [20, 21]. The electrospun fibrous scaffolds are three-dimensional (3D) constructs, designed like ECM, to support cell attachment, proliferation, migration, and differentiation, promote cell secretion of bioactive molecule, and accelerate functionality formation of tissues or organs. And the disorder or regular nanofibers can meet requirements of different tissues. The high porosity and specific surface area of electrospun fibers enable them as the useful drug carriers for inhibiting diseased cells and tissue engineering scaffolds for cells growth, leading to the complete regeneration [22]. Therefore, drug-loaded electrospun fibrous scaffolds have good potential as drug carriers and cell growth scaffolds.

**Figure 1:**
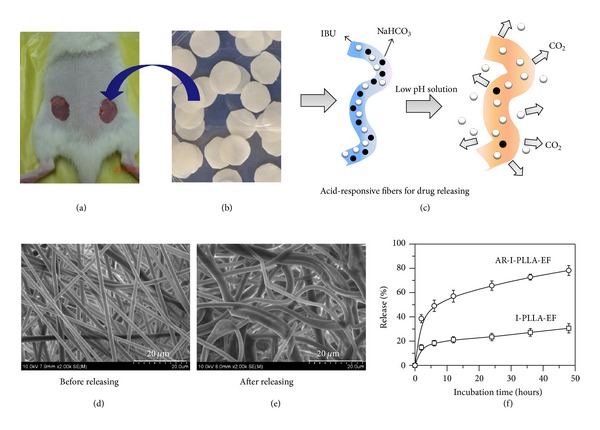
(a) Photograph of a rat model; (b) implantable electrospun fibrous scaffolds; (c) schematic illustration of acid-responsive electrospun fibers; (d) AR-I-PLLA-EF before releasing; (e) AR-I-PLLA-EF after releasing in pH 5.0; (f) the cumulative release curve of drug.

Consideration of the bacterial infection can cause the surrounding microenvironment to become acidic [23]; we designed a feasible acid-responsive controlled release systems as repairing tissue scaffolds, which are used to intelligently regulate the anti-inflammatory agent release with the change of acid microenvironment in regions where the pH is reduced below 7.4, leading to a good restrain of inflammation on the early stage and a scarless reparation on later stage. ibuprofen (IBU) was applied as the model drug (Figure 1). The results may cast some highlight on the relationship between inflammation and scars, developing the novel drug-loaded biomaterials for scarless healing.

## 2. Materials and Methods

### 2.1. Materials

Poly(L-lactide) (PLLA, Mw = 100 kDa, Mw/Mn = 1.6) was purchased from Sigma company. Dichloromethane (DCM), sodium bicarbonate (SB, NaHCO_3_), and N, N-dimethylformamide (DMF) were of reagent grade and were purchased from Sinopharm Chemical Reagent Co., Ltd. All other chemicals and solvents were of reagent grade or better and were purchased from GuoYao Regents Company (Shanghai, China), unless otherwise indicated.

### 2.2. Preparation of Electrospun Fibrous Scaffold

Electrospinning was carried out according to our previous report [24]. 1.0 g PLLA was completely dissolved in 4 g DCM and 2 g DMF for electrospun PLLA fibers (PLLA-EF). To prepare ibuprofen-loaded PLLA electrospun fibrous membranes (I-PLLA-EF), 1 g PLLA and 40 mg IBU were completely dissolved in a mix-solvent containing 4 g DCM and 2 g DMF for electrospinning. For acid-responsive IBU-loaded electrospun fibers (AR-I-PLLA-EF), 1 g PLLA and 40 mg IBU were dissolved in 4 g DCM and 2 g DMF; then 0.12 mL NaHCO_3_ saturated water (96 mg/mL) solution was added into the IBU/PLLA solution slowly with vigorous stirring for 30 min with magnetic stirrers. The blended solvent was then put in a syringe pump that was attached to the high voltage device. A negative electrode blanketed with aluminum foil was used as the collector plate.

### 2.3. Characterization and In Vitro Drug Release

Environmental scanning electron microscopy (ESEM, FEI, QUANTA250, The Netherlands) was used to investigate the morphology of the electrospun fibers. The electrospun fibrous scaffolds (weight, 100 mg) were simultaneously immersed in centrifuge tubes containing 25 mL phosphate-buffered solution (PBS, pH = 7.4) or sodium acetate-acetic acid buffer solution (pH = 5.0). The samples were incubated at 37°C for 48 h with mild shaking (100 rpm). At predetermined time intervals, 1 mL buffer solution was collected and replaced with 1 mL fresh buffer solution. The amount of released drug in the collected medium was determined by UV-vis spectroscopy at 224 nm. A standard calibration plot of IBU in the concentration range of 0–0.05 mg/mL was used to determine the concentration of the drug released. The percentage of the released drug was then calculated based on the initial weight of drug incorporated in the electrospun scaffolds.

### 2.4. Animal Model

All the procedures followed the policies of Shanghai Jiao Tong University School of Medicine and the National Institutes of Health. All animals were from the Animal Breeding Center, Shanghai Sixth People's Hospital Affiliated to Shanghai Jiao Tong University, School of Medicine, China. Male 8-week Sprague-Dawley rats (*n* = 40) weighing 200–250 g were housed one per cage with free access to food and water, in a room with controlled humidity and temperature (22°C), on a 12 h light/dark cycle. The rats were allowed to acclimatize for one week before the experiment.

The animals were divided into two groups (20 rats in each group) and anesthetized using ketamine (1 mL/kg body weight). A circular full-thickness 2 cm diameter skin wound extending through the panniculus carnosus was aseptically made on the dorsum on each side of midline (Figure 1(a)). In group one, the right lesion was untreated with any scaffolds (control group) and the left lesion was treated with electrospun fibrous scaffolds (PLLA group). In group two, the left lesion was treated with ibuprofen-loaded electrospun fibrous scaffolds (I-PLLA-EF group) and the right lesion was treated with stable acid-responsive ibuprofen-loaded electrospun fibrous scaffolds (AR-I-PLLA-EF group). The animals in each group were daily evaluated but euthanized on the 3, 7, 14, and 21 days (*n* = 5) after operation. Skin tissue samples from the wound site were excised in full depth and bisected for morphologic observation and molecular biological analysis.

### 2.5. The Examination of Wound Closure Rate

In order to determine the wound healing rate, the open wounds were drawn on graph acetate paper with a marker pen on the 3rd, 7th, 14th, and 21st days and photographed with a digital camera. The surface area of wound was measured with a planimetric program on computer by scanning the acetate sheets. The rate of wound closure was calculated using the following formula:

(1) Wound  closure  rate =[(Original  wound  area−Open  area  on⁡  final  day)Original  wound  area]  ×100%.

The original wound area is the initial wound area at day 0.

### 2.6. Histological Examination

Histological examination was performed to evaluate the quality of the regenerated tissue. Specimens were fixed in 10% formalin phosphate-buffered saline, dehydrated in ethanol, cleared in xylene, and in order arrangement embedded in paraffin. The above-treated specimens were then sectioned in 5 mm and stained with hematoxylin and eosin (HE) for histological observation. Further, Masson's trichrome stain was performed for observation of the collagen framework. As part of the histological evaluation, all slides were examined by a pathologist under a light microscope, without knowledge of the previous treatment.

### 2.7. Immunohistochemistry Staining

The immunohistochemical staining of bFGF and *α*-smooth muscle actin (*α*-SMA) were conducted by respective antibody. Sections were dewaxed with xylene and hydrated in decreasing concentrations of EtOH; endogenous peroxidase was blocked with 3% hydrogen peroxide for 10 min; nonspecific binding was blocked with 1% bovine serum albumin (BSA) for 30 min. Tissue sections were processed in 10 mM citrate buffer (pH 6.0) and heated to 100°C for 10 min for antigen retrieval. Primaries were applied for bFGF (1 : 100) and *α*-SMA (1 : 100) overnight at 4°C. Biotinylated secondary antibodies were applied at 1 : 200 for 30 min. Color development was performed with DAB for 3 to 5 min for all samples, followed by haematoxylin counterstaining.

### 2.8. Protein Isolation and Western Blot Analysis

To extract the whole protein fraction, frozen tissues were homogenized in the lysis buffer [50 mM Tris pH 7.4, 150 mM NaCl, 1% Triton-X 100, 0.5% deoxycholic acid, 0.1% SDS, and Protease-Inhibitor-Cocktail (1 : 100 v/v; Sigma)]. After incubation for 30 min on ice, the samples were centrifuged for 15 min at 12,000 r.p.m., at 4°C, and the supernatant was transferred to a new tube as whole protein fraction. Protein concentrations were determined using the Bio-Rad protein assay (Bio-Rad Laboratories, Hercules, CA) with BCA (Thermo Scientific, Waltham, MA) as standard. Then 20 *μ*g of proteins of each sample was electrophoresed using 12% sodium dodecyl sulfate polyacrylamide gel electrophoresis (SDS-PAGE) and then transferred to a polyvinylidene fluoride membrane (Millipore, Bedford, MA) which was blocked with 5% skim milk in Tris-buffered saline- (TBS-) T (10 mM Tris, 150 mM NaCl, and 0.1% Tween-20) for 1 hour at room temperature. The membrane was incubated with the primary antibody for 2 hours and was washed several times with TBS-T, and then it was incubated with a horseradish peroxidase conjugated secondary antibody for 1 hour. Finally, the membrane was washed and developed with an enhanced chemiluminescence detection system (Millipore, Bedford, MA). Signal intensity was quantified by image analyzer (Image J). The primary antibody used in this study was anti-*α*-SMA (1 : 500; Proteintech, Chicago, USA). GAPDH was analyzed as internal control with anti-GAPDH antibody (1 : 1500; Thermo Scientific, Waltham, MA).

### 2.9. Quantitative Real-Time PCR Analysis

After the skin wound was treated with different scaffolds or negative control for 14 days, total RNA from the regenerate tissues was prepared using Trizol Reagent (Thermo Scientific, Waltham, MA) according to the manufacturer's instructions. The integrity of RNA was verified by optical density absorption ratio OD260 nm/OD280 nm between 1.8 and 2.0. cDNA synthesis was conducted according to the RNA PCR kit protocol (Thermo Scientific, Waltham, MA). The primer sequences are shown in Table 1. Quantitative polymerase chain reaction (qPCR) amplification was performed in triplicate, using the SYBR Green PCR reagent system. Real-time quantitative PCR was carried out as follows: initial denaturation for 10 min at 95°C and 40 cycles of PCR consisting of 15 sec at 95°C and 45 sec at 60°C. Quantifications were always normalized using endogenous control GAPDH. Relative quantification was performed according to the comparative 2^−ΔΔCt^ method as previously described [25].

### 2.10. Statistical Analysis

The data were expressed as the mean ± standard deviations (SD). Statistical significance was determined with the student's *t*-test when there were two experimental groups. Multiple comparisons between the groups were performed with one-way analysis of variance (ANOVA) and post hoc tests (SAS 9.1 for Windows).* P* value <0.05 was statistically considered to be significant. All experiments were performed three times.

## 3. Results

### 3.1. Characterization and In Vitro Drug Release

The key component of these drug carriers is sodium bicarbonate (NaHCO_3_), which can be incorporated into electrospun fibers by using coelectrospinning. As described by Ke et al. [26], NaHCO_3_ reacted with acidic solution and the CO_2_ quickly released out of the fibers when fibrous scaffolds were incubated into acidic solution, which accelerated drug release from the inner sections of the fibers.

Morphology of all electrospun fibrous scaffolds showed no beads in the fibrous structure, the fibers were uniform in size and randomly interconnected (Figure 1(d)), and all samples were further tested by SEM after incubation for 48 h at pH 5.0 (Figure 1(e)). Compared with the original formation (Figure 1(d)), the 3D structure of electrospun fibrous scaffolds after incubation for 48 h at pH 5.0 maintained a 3D fibrous structure, even though fibers were swollen slightly compared with the original formation shown in Figure 1(d). The in vitro release profiles of IBU-loaded electrospun fibrous scaffolds in pH 5.0 solutions were shown in Figure 1(f). The total amount of released drugs was around 30.6% and 78.2% of I-PLLA-EF and AR-I-PLLA-EF after incubation for 48 h in pH 5.0 buffer solution, respectively. The release rate of the loaded IBU was accelerated with NaHCO_3_ in fibers in the acidic solution.

### 3.2. Analysis of Skin Wound Closure in the Rat Models

**Figure 2:**
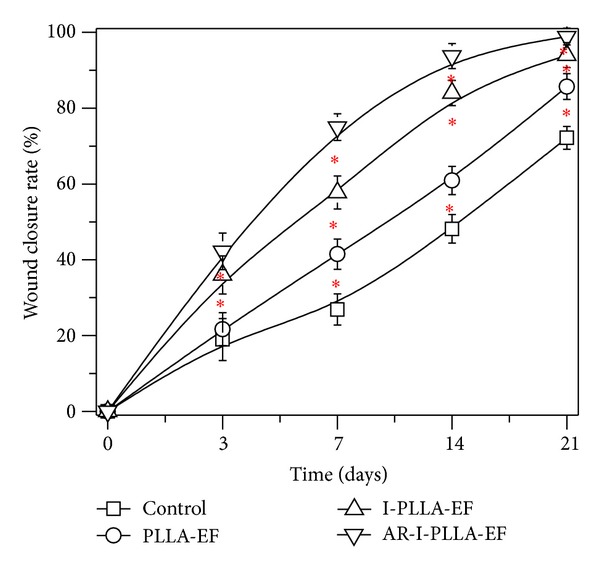
The wound closure rate of wound healing in the skin wound repair. Error bars represent SD. Wound healing rates of SD rats compared with control group; *P < 0.05.

Wound size in all groups was recorded on days 0, 3, 7, 14, and 21 after operation and expressed as percentage of the initial wound area (Figure 2). The wound area significantly decreased in all groups 7 days after skin excision. The residual wound area on the 7th day in control group was 73.11% and they were 58.52%, 42.25%, and 25.00% in PLLA group, I-PLLA-EF group, and AR-I-PLLA-EF group, respectively (*P* < 0.05). In addition, major differences happened on the 14th day after skin excision with the residual wound area being 51.84%, 39.06%, 16.00%, and 6.25%, respectively, in the same sequence as above; these differences were statistically significant (*P* < 0.05).

### 3.3. Histological Examination of Inflammatory Reaction and Scar Formation

**Figure 3:**
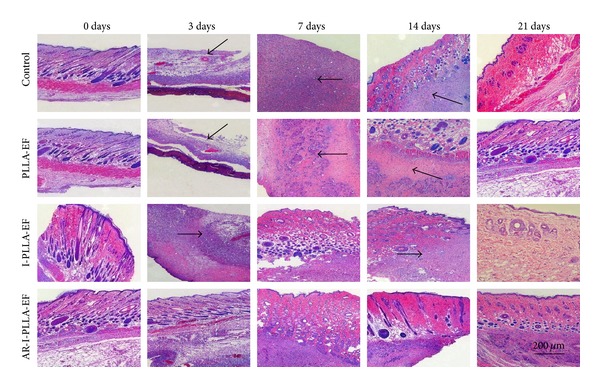
H&E staining of wounded skin repair after 3–21 days (×50).

Histologic analysis was performed to determine the cellular effects on wounds treated with different fibrous scaffolds. The scaffold instantly adhered to the wound and absorbed the exudate. We found that the scaffolds integrated to the new tissues and showed degradation with time. They were not degraded on the 3rd day after operation but on the 21st day, and all types of the scaffolds implanted into the subcutaneous tissues had degraded and disappeared. As shown in Figure 3, three days after operation, the epidermis and dermis showed deficiency on the area of the wound, left subcutaneous tissues only. The inflammation was observed in every group in the subcutaneous tissues and appeared in the intact tissues around the wound as well. Inflammatory cells were observed in dermis of the surrounding areas, and proliferation of fibroblasts and mild hemorrhage were seen. During this inflammatory phase of wound healing, the IBU-loaded groups showed a lighter degree of infiltration of inflammatory cells; especially in AR-I-PLLA-EF group, the control of inflammatory reaction was better than I-PLLA-EF group. Especially on the 7th day, in AR-I-PLLA-EF group, the surface of epidermis became even. After 21 days in AR-I-PLLA-EF group the defect had almost disappeared and the wound was filled with fibroproliferative tissue. The surface of the wounds was covered with new epithelium.

**Figure 4:**
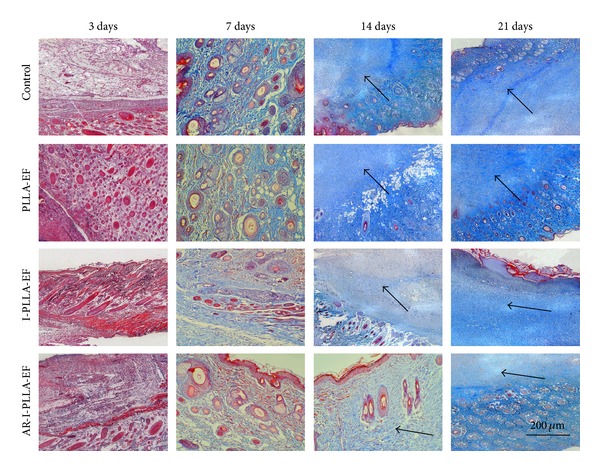
Masson's trichrome stained histological sections after 3–21 days (×50).

Masson's trichrome stained histological sections of the wounded skin samples showed more collagen content (blue staining) in control group and PLLA group than the IBU-loaded two groups with time (Figure 4). And on the 21st day after operation, the collagen content of AR-I-PLLA-EF group was less than other three groups.

### 3.4. Immunohistochemical Examination of bFGF and *α*-SMA Expression

**Figure 5:**
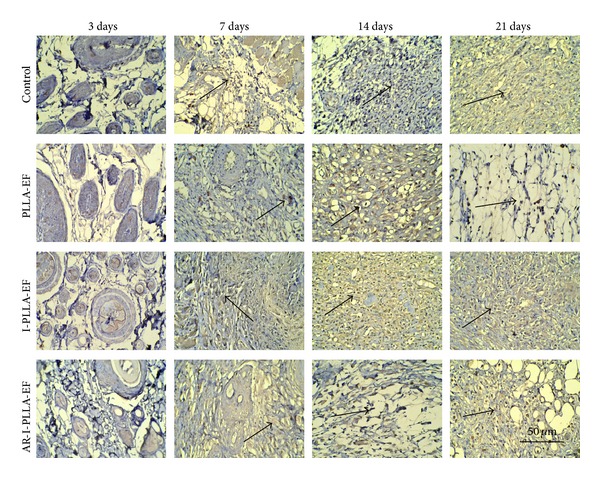
Wound skin repair operation after 3–21 days for immunohistochemical detection of bFGF (×200).

To evaluate bFGF expressions in situ, the immunohistochemistry approach was used. In I-PLLA-EF group and AR-I-PLLA-EF group immunohistochemistry revealed significant staining of bFGF (Figure 5). At postwounded day 3, bFGF expression was present in four groups with the weak staining but with a relatively higher expression in AR-I-PLLA-EF group. On the 7th day after operation, the expression of bFGF was increased in all groups and it was still higher in AR-I-PLLA-EF group than others. The significant difference on the 14th day was that the expression of bFGF showed strong positive staining with large area in AR-I-PLLA-EF group. This superiority of AR-I-PLLA-EF group lasted to the 21st day after operation with a slightly higher expression than the 14th day.

**Figure 6:**
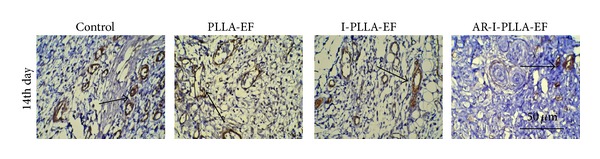
Immunohistochemical detection of *α*-SMA after 14 days of the operation (×200).

Sections from the wound showed *α*-SMA expression in skin wounds after 14 days of the operation (Figure 6). The positive staining of *α*-SMA in control group was the strongest and decreased in proper order successively in PLLA-EF group, I-PLLA-EF group, and AR-I-PLLA-EF group. The expression of *α*-SMA in AR-I-PLLA-EF group was less than other three groups.

### 3.5. Western Blot Analysis

**Figure 7:**
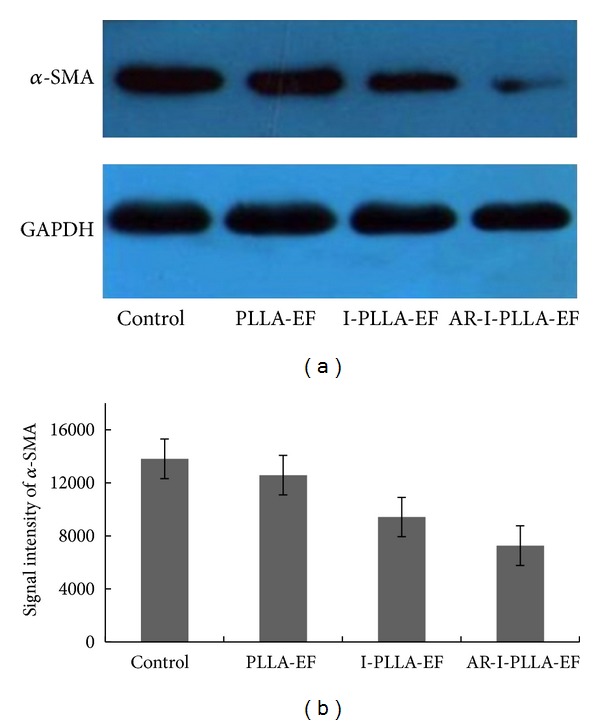
Western blot analysis of *α*-SMA on the 14th day after operation. (a) The levels of *α*-SMA in scars of different groups by Western blot. (b) Analysis of the signal intensity of *α*-SMA was performed among four groups. *P < 0.05 compared to control group; **P < 0.01 compared to control group.

Western blot analysis showed that levels of *α*-SMA expression decreased in IBU-loaded groups relative to the non-IBU-loaded groups (Figure 7). The expression level of *α*-SMA in control group was the highest and decreased in PLLA-EF group, I-PLLA-EF group, and AR-I-PLLA-EF group, respectively. The expression of *α*-SMA in AR-I-PLLA-EF group was the least among four groups.

### 3.6. qRT-PCR Analysis

**Figure 8:**
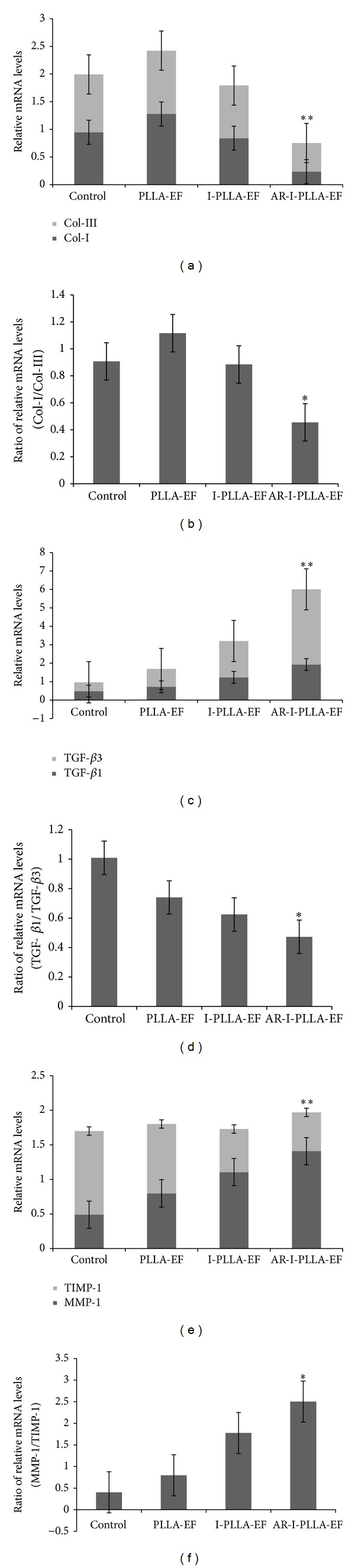
qRT-PCR analysis of the relative mRNA levels of collagen I and collagen III (a); TGF-*β*1 and TGF-*β*3 (c); MMP-1 and TIMP-1 (e), **P < 0.05 compared to control group. The ratio of collagen I/collagen III (b); TGF-*β*1/TGF-*β*3 (d); MMP-1/TIMP-1 (f) for 14 days after operation, using relative mRNA levels through qRT-PCR analysis, *P < 0.05 compared to control group.

On the 14th day, the relative expression of collagen I (Col-I) and collagen III (Col-III) of AR-I-PLLA-EF group was significantly different from the control group (*P* < 0.05) as shown in Figure 8(a). The expression of Col-I of AR-I-PLLA-EF group was the lowest in the four groups with more than four times lower than control group, and the expression of Col-III of AR-I-PLLA-EF group was the lowest in the four groups as well, about two times lower than the control group. Though the relative expressions of Col-I and Col-III of AR-I-PLLA-EF group were both the lowest among the four groups, with a relative higher expression of Col-III than that of Col-I, the ratio of Col-I/Col-III of AR-I-PLLA-EF group was still lower than control group, almost two times lower as shown in Figure 8(b) (*P* < 0.05). Interestingly, the relative expressions of Col-I and Col-III of PLLA group were somewhat higher than control group, though there were no significant statistical differences between them, maybe owing to the stimulation to collagen deposition of PLLA scaffold without IBU.

As shown in Figure 8(c), the relative expression of TGF-*β*1 and TGF-*β*3 was increased from control group, PLLA group, and I-PLLA-EF group to AR-I-PLLA-EF group in turns, respectively. And there were significant statistical differences between control group and AR-I-PLLA-EF group, with the expression of TGF-*β*1 of AR-I-PLLA-EF group being almost four times higher than control group and the expression of TGF-*β*3 of AR-I-PLLA-EF group being even more than eight times higher than control group (*P* < 0.05). Because of the relative higher expression of TGF-*β*3 than TGF-*β*1, the ratio of TGF-*β*1/TGF-*β*3 of AR-I-PLLA-EF group was lower than control group as shown in Figure 8(d) (*P* < 0.05).

From the control group, PLLA group, and I-PLLA-EF group to AR-I-PLLA-EF group, the relative expression of MMP-1 was increased and the relative expression of TIMP-1 was decreased as shown in Figure 8(e). So the ratio of MMP-1/TIMP-1 was increased in turn as displayed in Figure 8(f). The expression of MMP-1 of AR-I-PLLA-EF group was almost three times higher than control group and the expression of TIMP-1 of AR-I-PLLA-EF group was more than two times lower than control group (*P* < 0.05). And the ratio of MMP-1/TIMP-1 of AR-I-PLLA-EF group was more than six times higher than control group (*P* < 0.05).

## 4. Discussion

Ideal wound treatments should accelerate healing and reduce scar complications. However, scarless healing is difficult in adult mammalian tissues. Accordingly, increasing attention has been directed to screening and developing products that are effective in both accelerating wound healing and preventing scars [26]. In some pathological conditions such as malignant tumor, infection, or inflammation, the pH around the lesions may reduce below 7.4 [27]. One research put forward evidence that during the early fermentation period the bacterial abundance increased quickly and the pH values concurrently decreased rapidly without any initial pH increase [28]. Another study also displayed that, after controlling the infection of bacteria, the pH of or around the lesion area increased [29]. And the alkaline pH can increase both bacteriostatic and bactericidal activities of both planktonic and biofilm-associated bacteria [30]. Therefore, the microenvironment around bacteria is inclined to acid, which is in favor of the growth and proliferation of bacteria. Because of excessive inflammation, which resulted in inappropriate deposition of extracellular matrix which can lead to overgrowth, hardening, and/or scarring of tissues [31], we structured the acid-responsive ibuprofen-loaded PLLA fibrous scaffolds to control the excessive inflammation, hoping to achieve complete regeneration without scar.

In our histochemistry studies, ibuprofen effectively controlled the excessive inflammation in the early stage of the healing process, and the inflammation of AR-I-PLLA-EF group was even lighter than I-PLLA-EF group, which because of the accelerating release of ibuprofen resulted from the NaHCO_3_. Besides, ibuprofen can block the biosynthesis of inflammatory prostaglandins, especially prostaglandin E2 [32, 33]. Therefore, ibuprofen can alleviate postoperative pain and itch to some extent as well, which may benefit the peaceful healing process, owing to the reduction of the scratching frequency, especially for children.

Except that fetal skin wounds healing does not mount an inflammatory response after injury, fetal wounds differ from adult wounds also in extracellular matrix (ECM) components, growth factor expression and cellular differences, and so forth [34]. Changing one of these reactions may result in scarless healing in adult mammals. Our Masson's trichrome stained histological sections showed more collagen deposition in control group and PLLA group than the IBU-loaded two groups with time. Further through qRT-PCR analysis, we found that the relative expression of Col-I and Col-III of AR-I-PLLA-EF group was significantly different from the control group (*P* < 0.05) as shown in Figure 8(a); the expressions of Col-I and Col-III of AR-I-PLLA-EF group were both the lowest in the four groups, while, with the relative higher expression of Col-III than that of Col-I, the ratio of Col-I/Col-III of AR-I-PLLA-EF group was still lower than control group as shown in Figure 8(b) (*P* < 0.05). These results coincided with other studies [7, 17]. bFGF, which is a potent mitogen and chemoattractant for endothelial cells, fibroblasts, and keratinocyte can stimulate the metabolism, growth of the ECM, and the movement of mesodermally derived cells [35]. In our immunohistochemical examination, the staining for bFGF of AR-I-PLLA-EF group was stronger and the area of staining was larger than other groups as shown in Figure 5. This is in line with the study of Shi et al. [19] where the bFGF had antiscar effects on wound repair in vitro and in vivo, though other studies thought that decreased FGF activity is associated with scarless wound healing [36]. In addition, *α*-SMA is critical for wound contraction [16] and plays a major role in fibrosis as well, as it sustained the myofibroblasts to form the granulation tissue [36], which inevitably leads to fibrotic scar tissue formation. From our immunohistochemical examination and western blot analysis, the expression level of *α*-SMA of AR-I-PLLA-EF group was the lowest among four groups; the potential of forming scars of AR-I-PLLA-EF group, to some degree, was less than other groups.

There were lots of studies focused on TGF-*β* and the expression ratio between TGF-*β*1/2 and TGF-*β*3 during the scarless healing [8, 37]. And further studies found that, when TGF-*β*3(−/−) fetal mice wounded, the wounds healed with scarring, and, to the contrary, when TGF-*β*3 was administered in adult wounds, the scars were reduced [38, 39]. As fetal skin possesses the ability to regenerate completely, adults with scarless healing may need higher ratios of TGF-*β*3 to TGF-*β*1/2, and our results are consistent with the above studies. Other important molecules in complete healing were MMPs and TIMPs. Like other studies which implied that there were high levels of MMPs expression in dermal fibroblasts from nude mice [40] and scarless wounds have a higher ratio of MMP to TIMP, which may be due to the decreased expression of TGF-*β*1, as it decreases MMP and increases TIMP expression, favoring collagen accumulation and scarring [41], our studies showed that, from the control group, PLLA group, and I-PLLA-EF group to AR-I-PLLA-EF group, the relative expression of MMP-1 was increased and the relative expression of TIMP-1 was decreased as shown in Figure 8(e); the ratio of MMP-1/TIMP-1 was increased in turn as displayed in Figure 8(f). All these findings implied a complete and scarless healing.

## 5. Conclusion

A novel acid-responsive ibuprofen-loaded electrospun fibrous scaffold was easily fabricated based on NaHCO_3_ for antiscar skin healing. In general, the results of this study demonstrate that the acid-responsive ibuprofen-loaded electrospun fibrous scaffolds can prevent excessive inflammation in the early stage of the wound healing as drug carriers and provide scaffolds for cells to regenerate the achievement of a complete and scarless healing in the later stage.

## Figures and Tables

**Table 1:** Different gene specific primers.

	Sense	Antisense
Collagen I	5′-TGTGTTGCTGAAAGACTACC-3′	5′-TAGCACCAGAAATTCCTTCC-3′
Collagen III	5′-GTCCACAGCCTTCTACAC-3′	5′-TCCGACTCCAGACTTGAC-3′
TGF-*β*1	5′-AAGGACCTGGGTTGGAAGTG-3′	5′-TGGTTGTAGAGGGCAAGGAC-3′
TGF-*β*3	5′- GCACAGAGCAGAGAATCG-3′	5′-CTCCATTGGGCTGAAAGG-3′
MMP-1	5′-AGACCATAGTGACAATAACC-3′	5′-AACATTAGTGCTCCTACATC-3′
TIMP-1	5′-ACACGCTAGAGCAGATAC-3′	5′-CACAGCTACAGGCTTTAC-3′
GAPDH	5′-GTCGGTGTGAACGGATTTG-3′	5′-TCCCATTCTCAGCCTTGAC-3′
